# Compensation of subjects for participation in biomedical research in resource – limited settings: a discussion of practices in Malawi

**DOI:** 10.1186/s12910-019-0422-6

**Published:** 2019-11-14

**Authors:** Wongani Nyangulu, Randy Mungwira, Nginanche Nampota, Osward Nyirenda, Lufina Tsirizani, Edson Mwinjiwa, Titus Divala

**Affiliations:** 1 Public Health Nutrition Research Group (PHNG), College of Medicine, Blantyre, Malawi; 2 Blantyre Malaria Project, University of Malawi College of Medicine, Blantyre, Malawi; 3 Malawi-Liverpool Wellcome Trust Clinical Research Programme, Blantyre, Malawi; 4 Dignitas International, Zomba, Malawi; 5 Helse Nord Tuberculosis Initiative, University of Malawi College of Medicine, Blantyre, Malawi; 6 London School of Hygiene & Tropical Medicine, Keppel Street, Bloomsbury, London, WC1E 7HT UK

**Keywords:** Compensation, Biomedical research, Research participant, Coercion, Undue influence, Exploitation

## Abstract

**Background:**

Compensating participants of biomedical research is a common practice. However, its proximity with ethical concerns of coercion, undue influence, and exploitation, demand that participant compensation be regulated. The objective of this paper is to discuss the current regulations for compensation of research participants in Malawi and how they can be improved in relation to ethical concerns of coercion, undue influence, and exploitation.

**Main text:**

In Malawi, national regulations recommend that research subjects be compensated with a stipend of US$10 per study visit. However, no guidance is provided on how this figure was determined and how it should be implemented. While necessary to prevent exploitation, the stipend may expose the very poor to undue influence. The stipend may also raise the cost of doing research disadvantaging local researchers and may have implications on studies where income stipend is the intervention under investigation. We recommend that development and implementation of guidelines of this importance involve interested parties such as the research community and patient groups.

**Conclusion:**

Compensating human research subjects is important but can also act as a barrier to voluntary participation and good research efforts. Deliberate measures need to be put in place to ensure fair compensation of research participants, avoid their exploitation and level the field for locally funded research.

## Background

On 01 November 2017, the Malawi government through the National Health Sciences Research Committee (NHSRC), directed that all human subjects research should provide study participants with US$10 per study visit as compensation for costs. To our knowledge, Malawi does not have a comprehensive document which provides a breakdown of how the new US$10 compensation was determined or how it should be implemented. In 2018, Malawian researchers and regulators proposed a standardized approach for implementing the compensation for research subjects, but this is yet to be adopted by the NHSRC [1].

Compensation of research participants is a common and widely acceptable practice [2]. Compensation may be handed out as refunds for expenses incurred by participants; for time, effort and inconvenience; injury or harm associated with research participation or as incentives to stimulate participants to follow the study protocol to completion [2]. However, this practice raises ethical concerns of coercion, undue influence, and exploitation [3], hence the need for regulation.

In the context of human biomedical research, there are no universally agreed definitions for coercion or undue influence; nor is there agreement on how much compensation constitutes undue influence [3, 4]. According to the Belmont Report, “***Coercion*** *occurs when an overt threat of harm is intentionally presented by one person to another in order to obtain compliance.* ***Undue influence****, by contrast, occurs through an offer of an excessive, unwarranted, inappropriate or improper reward or other overture in order to obtain compliance. Also, inducements that would ordinarily be acceptable may become undue influence if the subject is especially vulnerable*” [5]. Coercion and undue influence are rightfully considered due to their effect on informed consent [2, 4].

In order to participate in biomedical research, participants must voluntarily agree after fully considering all the risks and benefits of participation. If large enough, there is concern that compensation may induce potential participants to participate in research which conflicts with their deeply held values and beliefs or overlook certain risks which they would otherwise not accept, had the ‘compensation’ not been present [4]. This would be a violation of the informed consent process, which is itself an application of the principle of autonomy or respect for persons; the emphasis of which follows a historical record of abuse and mistreatment of research participants [5].

Similarly, concerns that compensation may cause undue influence must be weighed against the risk of exploitation and the ability of participants to make informed choices as autonomous agents. While some scholars argue that participants should enroll in research studies for altruistic reasons [6], many more support compensation of participants [3], with some arguing whether participants are compensated enough [7]. In addition, evidence suggests that while the amount of compensation affects willingness to participate in research, it does not minimize the ability of individuals to reasonably assess risk of participation in studies [8–10]. In this paper, we discuss compensation practices in Malawi. We first take an international perspective on compensation guidelines for low resource settings. We review compensation guidelines in Africa using South Africa as a proxy. Then, we focus on the merits and drawbacks of current Malawian regulations, propose changes to these regulations and make recommendations to improve on current practice.

### Main text

#### International research perspective

Low and middle-income countries (LMICs) especially in Sub-Saharan Africa have long been fertile ground for research on infectious diseases which are highly prevalent in these settings [11]. However, the global spread of the Human Immunodeficiency Virus (HIV) in the 80s and 90s which disproportionately affected these regions [12] led to a larger increase in the disparate burden of biomedical research from wealthy developed countries to developing nations. Increasingly, the poor and citizens of low and middle-income countries in Africa, South America and Asia became subjects of research funded by stakeholders in North America and Western Europe [13–16]. To ensure ethical conduct of clinical research in these low socio-economic groups and regions, international guidelines were developed to aid researchers to navigate this new landscape and protect participants from exploitation and harm [17].

The revised Council for International Organizations of Medical Sciences (CIOMS) International Ethical Guidelines for Health-related Research Involving Humans published in 2016 are the latest in a series of guidelines published on how ethical principles should be applied in biomedical research in LMICs [17, 18]. They make a distinction between reimbursement (repayment for out-of-pocket expenses incurred) and compensation. The guidelines state that participants should be “reasonably reimbursed” for direct research costs such as transport to and from the study site. They should also be “reasonably compensated” for inconvenience and time spent. Monetary compensation should be proportional to the time spent and calculated using in-country hourly wage as a reference. Compensation can also be in the form of goods such as food packages and services such as healthcare packages and medical insurance but should never be “meant to compensate for risk”. There are also guidelines for compensation for persons unable to give consent, compensation after study withdrawal (including research related harms incurred during the study) and studies which use financial incentives as interventions [17].

#### Compensation and trial incentives for research participants in Africa

In Africa, there is no uniform approach to compensation for research subjects across various countries [19]. This reflects different legal and policy environments as well as cultural differences across the continent. We will briefly look at practices in South Africa before review and discussion of practices in Malawi.

The South African National Health Act (2003) makes compliance with guidelines for Good Clinical Practice (GCP) mandatory [20]. The second edition of the South African Guidelines for GCP in the conduct of clinical trials (2006) includes sections on the compensation of trial participants for research related injury and trial incentives. Incentives, defined as payments or concessions made to encourage a certain action or behavior, are acceptable under these guidelines. Incentives should not be excessive as to make an offer a participant “cannot refuse”. Participants must be reimbursed for all “reasonable costs incurred” during trial participation. Incentives can be financial, transport or food. Information on incentives must be provided to participants and included in the protocol and in the case of multicenter trials, differences in compensation across sites must be explained to the participants.

The guidelines also require sponsors to provide compensation for injury incurred due to participation in research. This is irrespective of whether the participant can prove negligence on the part of the sponsor or investigator. However, the sponsor is under no obligation to pay compensation where there is “failure of a medicinal product to have its intended effect or to provide any other benefit” and “injury caused by other licensed medicinal products…. for…. comparisons with the product under trial”. Furthermore, the sponsor may not pay compensation where there is a failure of placebo to provide therapeutic benefit and injury due to deviations from the agreed protocol, a third party or negligence on the part of the participant [20].

#### Compensation of research participants in Malawi

The National Health Sciences Research Committee is one of two research ethics committees (RECs) delegated by the Government of Malawi to provide research oversight and develop guidelines for the conduct of research in Malawi [21, 22].

On 01 November 2017, the NHSRC notified all investigators involved in health research on human subjects “to reimburse all study participants a Malawi Kwacha equivalent of 10 US $ as stipend to participants during each scheduled visit to the facilities where research is taking place” [23]. However, no formal guidelines were laid out on how this figure was determined, how this guideline would be implemented nor the specific purpose for which participants would be reimbursement. In 2018, researchers and regulators in Malawi proposed a standardized approach which used renumeration tables to calculate how much and for what purpose a participant should be compensated [1]. However, this has not yet been implemented.

Four models for compensation of research participants are generally recognized: market model, wage-payment model, reimbursement model and appreciation model [2, 24]. In the market model, forces of supply and demand determine the amount to be compensated and payment serves as an incentive for enrollment into the study or completion of follow up schedule. In the wage-payment model, participation in research is treated as a form of unskilled labor with a standardized wage for time and effort put into the research process. The wage is commensurate with the minimum wage in the country. The reimbursement model recognizes that participants must not incur a cost for participation in research. Participants are reimbursed for transport, meals, lodging and it may also take into account lost wages or economic opportunity for time spent in the study. In the appreciation model, payment is a reward or token of thanks for participation in the study [24]. Regarding guidance for compensation of research participants in Malawi, it is unclear which, if any, of these models the guideline to pay $10 stipend falls under.

We support compensation of research participants and believe that the $10 minimum compensation per study visit was set to prevent exploitation. While research provides evidence-based solutions to problems faced by mankind and can thus be a public good, it provides direct benefits to sponsors (e.g. pharmaceutical companies) and researchers. The pharmaceutical industry is worth billions of dollars and investigators and those they employ are paid for their work. It is only reasonable and fair that individuals who participate in research also receive direct benefits in addition to ancillary care provided in many research settings, and their communities have assured access to products post-registration. To do otherwise would be tantamount to exploitation of these participants especially in the context of research on the poor benefiting those that are well off. This benefit sharing is consistent with the principle of justice. This sentiment to expect reasonable and fair compensation is shared by community members [16].

However, to remain ethical, participant compensation need not be the sole reason for subjecting oneself to research procedures. In a setting where over 60% of the population lives on less than $2 per person per day, the current $10 per study visit, may easily act as undue influence. Poverty is an insidious barrier to autonomous decision-making, predisposing individuals to unfair power dynamics [15]. The principle of respect for persons requires that research participants are treated as autonomous individuals and those with diminished autonomy are protected [5].

The new research participant compensation strategy is likely to slow rather than nurture locally brewed research. Some researchers, especially the locally funded, and students, may not have the necessary financial resources to honor it. This would, in turn, suppress locally brewed research in favor of the well-funded, often international work. Although exceptions for payment of $10 stipend have been made for undergraduate and postgraduate students, a cohort of local graduate researchers not undertaking academic study are still required to adhere to guidelines. For the exempted, their recruitment efforts may be subject to unfair competition from well-funded studies that are compensating participants at a higher rate. Research regulation should nurture and not deter research efforts.

Lastly, the US$10 participant stipend may affect the interpretation of studies where outcomes are influenced by social-economic determinants and where financial incentives serve as interventions. For example, in a study to evaluate HIV care delivery models among adolescents where the primary outcome is retention in care, the inclusion of the stipend and other incentives may affect the results of the study by shifting the social-economic base. Therefore, effects of social economic determinants on retention can hardly be realistically measured. This creates a dilemma, particularly on how to strike a balance between science and ethics for these types of studies when the social-economic structure has been altered.

### Recommendations

#### Development of national guidelines on compensation of participants

Malawi has taken a significant step forward with development of regulations for research including addressing the important angle of participant compensation. However, the release of any regulation is incomplete if not accompanied by instructions for practical implementation by all relevant users. Clearer definitions of compensation, reimbursement, and incentives, their applicability and enforcement are urgently needed. If researchers, ethics committees, regulatory authorities, health institutions, and representatives of research participants (Community Advisory Boards and patient groups) are given detailed and clear instructions on what is expected of them in what circumstances of biomedical research, implementation of regulations would be smoother. We urge the NHRSC, and similar bodies elsewhere to consider developing such guidelines as they release new regulations.

#### Community consultation

Regulation for a collaborative process such as research, may achieve its goals better if developed collaboratively. The current regulation for participant compensation was handed down without any warning or consultation leading to disjointed implementation and adherence. We recommend timely consultations with the research community which, at a minimum, comprises of researchers, ethics committees, regulatory authorities, health institutions, and representatives of research participants (Community Advisory Boards and patient groups) and communities. Detailed consultations with prospective implementers and subject of regulation lends the process legitimacy and breathes success into the final outcome. In this direction, we welcome the consultative meeting the regulators had with researchers in 2018 and hope that more sections of the research community will also be reached out to and that outcomes of such meetings will be part of an amended regulation and guideline.

#### Alternative compensation

It has been proposed previously and we echo this sentiment that compensation or payment should not only be monetary but can also be in the form of goods (e.g. food, soap, educational materials etc.) and services such as “ancillary” care provided to participants and their families [2, 24]. This helps prevent monetization of the researcher-participant relationship but also prevents awkwardness in situations where financial compensation may not be the most appropriate method [25, 26]. It may also provide a reprieve for local researchers who may not afford monetary but alternative compensation. However, we are mindful that there may not be a one size-fits-all kind of compensation and that in some areas, monetary forms of compensation may be the best way. We propose adapting mode of compensation to community preferences determined through consultative meetings or qualitative studies.

## Conclusions

We welcome strides the Malawi government has made with respect to research regulation and appreciate the setting of minimum amount of money for human research subject compensation as a means to prevent exploitation. We are however concerned that the current amount of US$10 may: a) suppress the often-underfunded local researchers, and b) in some cases, fail to balance off coercion and undue influence especially being in a population where most do not make more than $2 per day. We are of the opinion that regulations development for a collaborative process like research can cover more ground and become more implementable if it involves the entire research community. Just like standard operating procedures accompany research protocols, new regulations ought to be released with clear guidelines showing expectations of all members of the research community which, at a minimum, comprises of researchers, ethics committees, regulatory authorities, health institutions, and representatives of research participants (Community Advisory Boards and patient groups) and local communities. We also recommend a slight departure from the one-size-fits-all approach for human subject research participant compensation towards a needs-responsive approach that takes community ideas onboard.

## Data Availability

N/A

## Abbreviations

CIOMS: Council for International Organizations of Medical Sciences
COMREC: College of Medicine Research and Ethics Committee
HIV: Human Immunodeficiency Virus
ICH-GCP: International Conference on Harmonization-Good Clinical Practice
NCST: National Commission for Science and Technology
NHSRC: National Health Sciences Research Committee
